# Resolution and Quantitation of Ribonucleosides and Deoxyribonucleosides in Digested Genomic DNA by UHPLC–MS/MS

**DOI:** 10.1002/bmc.70599

**Published:** 2026-08-13

**Authors:** Joshua J. Deppas, Reyna Jones, Robert A. Parise, Pinakin Pandya, Christopher J. Bakkenist, Jan H. Beumer

**Affiliations:** ^1^ Cancer Therapeutics Program UPMC Hillman Cancer Center Pittsburgh Pennsylvania USA; ^2^ Department of Pharmaceutical Sciences, School of Pharmacy University of Pittsburgh Pittsburgh Pennsylvania USA; ^3^ Department of Radiation Oncology, School of Medicine University of Pittsburgh Pittsburgh Pennsylvania USA; ^4^ Department of Pharmacology and Chemical Biology, School of Medicine University of Pittsburgh Pittsburgh Pennsylvania USA; ^5^ Department of Oncology Johns Hopkins University School of Medicine and Johns Hopkins Sidney Kimmel Cancer Center Baltimore Maryland USA

**Keywords:** ceralasertib, deoxynucleosides, LC–MS/MS, nucleoside quantitation, ribonucleosides

## Abstract

Deoxyuridine (dUrd) is often misincorporated into genomic DNA in cancer patients receiving antimetabolite chemotherapy. This activates base excision repair and recruits ataxia telangiectasia‐mutated and Rad3‐related (ATR), which coordinates DNA repair with nucleotide metabolism by facilitating ribonucleotide reductase (RNR) activity. Ceralasertib (AZD6738) is the most clinically advanced ATR inhibitor (ATRi) and may abrogate this regulatory pathway and diminish RNR‐mediated deoxynucleotide triphosphate synthesis. To further understand ATRi‐induced dUrd misincorporation, we developed a highly sensitive LC–MS/MS method to quantitate: deoxyuridine, uridine, guanosine, adenosine, cytidine, thymidine, deoxyguanosine, deoxyadenosine, and deoxycytidine from digested genomic DNA.

Assay application was demonstrated with genomic DNA digested from multiple murine cell lines treated with ceralasertib. Chromatographic separation was achieved with an Inertsil ODS‐3 (150 × 2.1 mm, 3 μm) column and a gradient elution program of 0.1% formic acid in water and 0.1% formic acid in methanol over an 18‐min run time. Detection was performed on a SCIEX 6500+ tandem mass spectrometer. The method proved to be accurate (90.8%–114.2%) and precise (< 7.73% CV) across analytes. Freeze–thaw stability (106.4%–114.3%), stability for 12 months at −80°C (88.7%–113.7%), and stability for 4 h at room temperature (104.1%–114.0%) were acceptable across QCs.

**Abbreviations**
5hmdC: 5‐hydroxymethyl‐2′‐deoxycytidine
5mdC: 5‐methyl‐2′‐deoxycytidine
Ade: adenosine
ATR: Ataxia telangiectasia‐mutated and Rad3‐related
ATRi: ATR inhibitor
BER: base excision repair
BrdU: 5‐bromo‐2′‐deoxyuridine
CDA: cytidine deaminase
CldU: 5‐chloro‐2′‐deoxyrudine
Cyt: cytidine
dAde: deoxyadenosine
dCyt: deoxycytidine
dGua: deoxyguanosine
dN: deoxyribonucleoside
dNTP: deoxynucleoside triphosphate
dTTP: deoxythymidine triphosphate
dUrd: deoxyuridine
dUrd: deoxyuridine
dUTP: deoxyuridine triphosphate
ESI: electrospray ionization
FBS: fetal bovine serum
Gua: guanosine
IS: internal standard
LLOQ: lower limit of quantitation
MRM: multiple reaction monitoring
QC: quality control
QCH: QC high
QCL: QC low
QCM: QC mid
rN: ribonucleoside
RNR: ribonucleotide reductase
Thy: thymidine
ULOQ: upper limit of quantitation
UNG: uracil‐DNA glycosylase
Urd: uridine
WT: wild type

## 1 Introduction

The presence of uracil in DNA is a result of the deamination of cytosine or the incorporation of deoxyuridine (dUrd) during DNA synthesis via DNA polymerases (Chakraborty and Stover 2020; Cui et al. 2019; Kuraoka et al. 2003). An elevated intracellular ratio of deoxyuridine triphosphate (dUTP) to deoxythymidine triphosphate (dTTP) (i.e., dUTP/dTTP) promotes dU misincorporation into DNA, which in turn activates base excision repair (BER) pathways (Ladner 2001). Misincorporation of dUrd is prevalent in most cancer patients receiving antimetabolic cancer chemotherapies (e.g., gemcitabine and 5‐FU), as these therapies are known to increase the intracellular dUTP/dTTP ratio (Guo et al. 2016; Noordhuis et al. 2004; Parker and Stivers 2011). To safeguard genomic integrity, uracil‐DNA glycosylase (UNG) mediates uracil recognition and excision from DNA (Mathews 2015). Cytidine deaminase (CDA), involved in free pyrimidine nucleotide metabolism, is upregulated in several cancer types and, under conditions of disrupted nucleotide metabolism, can contributed to elevated dUTP/dTTP and increase reliance on UNG‐mediated BER (Mathews 2015; Rajabpour et al. 2017; Scolaro et al. 2024). Excessive BER activity can generate numerous single‐strand breaks and destabilize replication forks, triggering recruitment of the ataxia telangiectasia‐mutated and Rad3‐related (ATR) protein kinase (Gohil et al. 2023). ATR activation coordinates DNA repair and cell cycle progression with nucleotide metabolism by activating ribonucleotide reductase (RNR) (Le et al. 2017; Nam and Cortez 2011), which catalyzes the reduction of ribonucleoside (rN) diphosphates to deoxyribonucleoside (dN) diphosphates. This process maintains adequate dTTP pools and preserves nucleotide balance, thereby preventing excessive genomic incorporation of dUTP. Therefore, when nucleotide biosynthesis is disrupted, the dUTP/dTTP ratio may increase, leading to genomic dU accumulation.

Small molecule ATR inhibitors (ATRi) can disrupt nucleotide homeostasis through inhibition of ATR‐CHK1‐dependent regulation of RNR‐mediated deoxynucleoside triphosphate (dNTP) synthesis, thus exacerbating replication stress and genomic instability (Le et al. 2017; Qiu et al. 2018). This disruption can elevate the dUTP/dTTP ratio, thus promoting the misincorporation of dUrd into the genome. Several ATRi are in clinical development, including ceralasertib (AZD6738), which is currently active in 30 clinical trials, including two Phase III clinical trials (clinicaltrials.gov access date 03/27/2026). To better understand ATR‐induced dUrd misincorporation into DNA, we developed and validated a specific and sensitive LC–MS/MS method capable of simultaneously quantitating rNs—uridine (Urd), adenosine (Ade), guanosine (Gua), and cytidine (Cyt) and dNs—deoxyuridine (dUrd), thymidine (Thy), deoxyadenosine (dAde), deoxyguanosine (dGua), and deoxycytidine (dCyt). The assay application was demonstrated with genomic DNA purified and digested from multiple murine cell lines. Specifically, wild type (WT) and UNG‐knockout YUMM1.7, MEF, and B16F10 cells were treated with ceralasertib. As these cells lines have been studied by our group and others, in the context of ATR and DDR inhibition previously, this was determined to be a biologically justified panel for our evaluation (Menolfi et al. 2018; Pandya et al. 2024; Proctor et al. 2021; Vendetti et al. 2023). Method development and validation were conducted in accordance with guidance by the US FDA on bioanalytical method validation (U.S. Department of Health and Human Services Food and Drug Administration 2018).

## 2 Experimental

### 2.1 Chemicals and Reagents

Adenosine (≥ 99% purity), cytidine (99% purity), guanosine (≥ 98% purity), uridine (≥ 99% purity), thymidine (≥ 99% purity), 2′‐deoxyuridine (≥ 98.5% purity), and 2′‐deoxyguanosine monohydrate (≥ 99% purity) were purchased from Sigma‐Aldrich (St. Louis, MO, USA). 2′‐Deoxyadenosine hydrate (≥ 99% purity) was purchased from ACROS Organics (Fairlawn, NJ, USA). [^13^C‐1,3‐^15^N_2_]‐2′‐Deoxyuridine (≥ 98.5% purity) and thymidine‐*α*,*α*,*α*,6‐d_4_ (≥ 99% purity) were purchased from CDN Isotopes (Quebec, Canada). [^13^C_5_]‐Adenosine (≥ 95% purity), [^13^C_5_;1′,2′;3′,4′,5′]‐cytidine (≥ 98% purity), [^13^C;^15^N_2_]‐guanosine (≥ 98% purity), and [^13^C;1,3;^15^N_2_]‐uridine (≥ 98% purity) were purchased from Toronto Research Chemicals (Toronto, Canada). [^13^C_10_;^15^N_5_]‐2′‐deoxyadenosine monohydrate (≥ 95% purity) and [^13^C_10_;^15^N_5_]‐2′‐deoxyguanosine hydrate (≥ 95% purity) were purchased from Cambridge Isotope Laboratories (Tewksbury, MA, USA). [^13^C;^15^N_3_]‐2′‐Deoxycytidine (> 98% purity) was purchased from Alsachim (Graffenstaden, France). Formic acid was purchased from Fisher Scientific (Fairlawn, NJ, USA). Methanol (HPLC grade) and water (HPLC grade) were purchased from Honeywell Burdick & Jackson (Charlotte, NC, USA). Nitrogen for mass spectrometric applications was purified with a Nitrogen Generator (Parker Balston, Haverhill, MA, USA).

### 2.2 Chromatography

The LC system consisted of a SCIEX (Framingham, MA, USA) ExionLC autosampler and 2− ad pumps flowing into an Inertsil ODS‐3 (150 × 2.1 mm, 3 μm) column using a gradient elution program. The temperature of the autosampler was set at 4°C, whereas the column oven was kept at 40°C. Mobile phase solvent A was 0.1% (*v/v*) formic acid in water, and mobile phase solvent B was 0.1% (*v/v*) formic acid in methanol. The initial mobile phase composition was 99.5% solvent A and 0.5% solvent B. From 0 to 10.0 min, at a 0.4‐mL/min flow rate, solvent B was increased to 7.0%. Between 10.0 and 12.0 min, solvent B increased to 15% and flow rate remained the same. From 12.0 to 12.1 min, solvent B increased to 60% and flow rate increased to 0.5 mL/min and conditions held until 14.0 min. At 14.1 min, solvent B was returned to initial conditions with a flow rate of 0.5 mL/min and maintained until 18.0 min. From 0 to 0.1 min and 13.0 to 18.0 min, the LC flow was diverted to waste.

### 2.3 Mass Spectrometry

Mass spectrometric detection was carried out using a SCIEX 6500+ mass spectrometer (Framingham, MA, USA) with electrospray ionization (ESI) in positive and negative‐ion, multiple reaction monitoring (MRM) mode. The mass spectrometric conditions and MRM *m/z* transitions monitored for ESI negative and positive analytes can be seen in Table S1 and Table S2, respectively. Control of the LC system and mass spectrometer, as well as data collection, was accomplished with Analyst software (version 1.7.2).

### 2.4 Preparation of Calibrators, Quality Controls, and Internal Standards

All analytes, stock solutions, internal standards (IS), and IS mixes were independently weighed and prepared initially with 50:50 HPLC‐grade water/methanol and stored at −80^o^ C. Thy, dGua, dAde, and dCyt were prepared at 2.0 mg/mL. Urd and dUrd were prepared at 1.0 mg/mL. Ade and Cyt were prepared at 1.0 mg/mL. Gua was prepared at 0.50 mg/mL. A master stock solution mix was then created to yield 1‐mM concentrations of Thy, dGua, dAde, and dCyt; 0.1‐mM concentrations of dUrd and Urd; and 0.01‐mM concentrations of Gua, Ade, and Cyt and stored at −80°C. On the day of assay, this mix was serially diluted (in steps of tenfold) in water to obtain the lower calibration working solutions. These working calibration solutions were used to produce seven calibrator levels (Table S3). For each calibration series, blank samples were also prepared in water. A master quality control (QC) stock solution mix was prepared independently from separate analyte weighings and stored at −80°C. This solution was diluted in water as previously described to produce the following QC concentration samples of either: Lower limit of quantitation (LLOQ), QC Low (QCL), QC Mid (QCM), and QC High (QCH). Analyte concentrations for each level can be seen in Table S4.

Thymidine‐*α*,*α*,*α*,6‐d_4_ (Thy‐IS), [^13^C_10_;^15^N_5_]‐2′‐deoxyguanosine hydrate (dGua‐IS), [^13^C_10_;^15^N_5_]‐2′‐deoxyadenosine monohydrate (dAde‐IS), [^13^C;^15^N_3_]‐2′‐deoxycytidine (dCyt‐IS), [^13^C‐1,3‐^15^N_2_]‐2′‐deoxyuridine (dUrd‐IS), and [^13^C;1,3;^15^N_2_]‐uridine (Urd‐IS) were prepared at 1 mg/mL. [^13^C_5_]‐Adenosine (Ade‐IS) and [^13^C_5_;1′,2′;3′,4′,5′]‐cytidine (Cyt‐IS) were prepared at 0.1 mg/mL. [^13^C;^15^N_2_]‐Guanosine (Gua‐IS) was prepared at 0.5 mg/mL.

A master IS mix was then prepared at 250 μM of dUrd‐IS and Urd‐IS; 100 μM of Thy‐IS, dGua‐IS, and dAde‐IS; and 15 μM of Gua‐IS, Ade‐IS, and Cyt‐IS. The master IS mix was then diluted tenfold in water and stored at −80°C. On the day of assay, a working IS mix was created by diluting the master IS mix 250‐fold in water to obtain final concentrations of 100 nM of dUrd‐IS and Urd‐IS; 40 nM of Thy‐IS, dGua‐IS, and dAde‐IS; and 5 nM of Gua‐IS, Ade‐IS, and Cyt‐IS.

### 2.5 Sample Preparation

Ten microliters of sample was added directly to a conical HPLC autosampler vial and diluted with 20 μL of working IS mix. Samples were vortexed for 30 s on a Vortex Genie‐2 set at 10 (Model G‐560 Scientific Industries, Bohemia, NY, USA) to ensure proper mixing. Finally, a volume of 20 μL was injected into the LC–MS/MS system.

### 2.6 Validation Procedures

#### 2.6.1 Calibration Curve and LLOQ

Calibration standards and blanks were prepared (see Section 2.4) and analyzed in triplicate to establish the calibration range with acceptable accuracy and precision. Standard curves were constructed individually by plotting the analyte‐to‐IS area ratio (response) versus the nominal concentrations in each sample. Standard curves were fitted by linear regression with weighting by 1/y^2^, followed by back‐calculation of concentrations. The deviations of these back‐calculated concentrations from the nominal concentrations were expressed as a percentage of the nominal concentration.

#### 2.6.2 Accuracy and Precision

The accuracy and precision of the assay were determined by analyzing samples at the LLOQ, QCL, QCM, and QCH in six replicates each in three analytical runs, together with independently prepared triplicate calibration curves. Accuracy was calculated at each test concentration as (mean measured concentration/nominal concentration) × 100%.

Assay precision was calculated using ANOVA, as previously described (Rosing et al. 2000), with SPSS 27.0 for Windows (SPSS Inc., Chicago, IL, USA). Back‐calculated concentrations of calibration and QC samples were entered with the run number as factor. From the resulting mean squares of the within runs and mean squares of the between runs, the intra‐assay and interassay precisions were calculated.

#### 2.6.3 Selectivity and Specificity

Although all analytes are analyzed separately, cross talk of each into the IS channel, and vice versa, was characterized by monitoring MRM channels. Each sample was separately spiked to 10× the upper limit of quantitation (ULOQ) or IS concentration followed by quantitation of response on other MRM channels. Carryover was assessed by injecting samples with 10× the ULOQ of each analyte, followed by serial blank injections.

#### 2.6.4 Extraction Recovery and Ion‐Suppression

Given that samples are directly added to HPLC vials and diluted with IS mix, there is no extraction of analyte required. Thus, no extraction recovery or ion‐suppression experiments were performed.

#### 2.6.5 Stability

Long‐term stability experiments were performed for master stock solution mixes and QCs. A freshly prepared master stock solution mix was compared against a master stock solution mix after storage at −80°C. Stability was expressed for each analyte as the percentage recovery of the stored solution (2 years) relative to fresh solution mix. In addition, the stability of a master stock solution mix at room temperature for 6 h was determined in triplicate.

QC stability testing was performed in triplicate at LLOQ, QCL, QCM, and QCH concentrations. The effect of 3 freeze/thaw cycles on analyte concentrations was evaluated by assaying samples after they had been frozen (−80°C) and thawed 3 times and comparing the results with those of freshly prepared samples. Long‐term QC stability was determined by assaying samples before and after storage (−80°C) for 12 months. To evaluate the stability of analytes in the autosampler (4°C), we reinjected QC samples and calibration curves approximately 72 h after the first injection and compared concentrations derived from the second injection against those from the initial injection (U.S. Department of Health and Human Services Food and Drug Administration 2018). Results from the second run were expressed as a percentage of their respective values from the initial run. Under typical conditions, the total volume in the HPLC vial is 30 μL with an injection volume of 20 μL, preventing us from performing any reinjections. Therefore, for this experiment, both sample volume and IS volume were doubled to yield a final volume of 60 μL, allowing for a second 20‐μL injection from the same HPLC vial.

#### 2.6.6 Dilutional Integrity

To verify dilutional integrity, the ability to dilute samples from above the ULOQ to within the validated concentration range, a stock solution mix of all analytes spiked above the ULOQ was first prepared and diluted within the assay range. The starting concentrations were as follows: 1000,000 nM of Thy, dGua, dAde, and dCyt; 100,000 nM of dUrd and Urd; and 10,000 nM of Gua, Ade, and Cyt. Samples (*N* = 3) were then diluted 100‐fold with water and assayed.

### 2.7 Assay Application

#### 2.7.1 Cell Lines

B16F10 Cas 9 WT control and B16F10 UNG‐knockout cells were created using lentivirus transduction and grown using D‐MEM that included 10% heat‐inactivated fetal bovine serum (FBS) and 1% Pen/Strep. To select for transduced B16F10 cells, media also included puromycin at a concentration of 1.5 μg/mL. YUMM1.7 Cas9 WT control and YUMM1.7 UNG‐knockout polyclonal cells were created with YUMM1.7 cells (Dr. Katherine Aird) via lentivirus transduction and grown using DMEM/F12 that included 10% heat‐inactivated FBS, 1% Pen/Strep, and MEM Nonessential Amino Acids. To select for transduced YUMM 1.7 cells, media also included puromycin at a concentration of 1.5 μg/mL. Mouse embryonic fibroblasts (MEF; WT 92Tag, UNG^−/−^ knockout 207Tag) were generated from 14.5‐day‐old embryos. Transfection was performed using an SV40 T‐antigen expression vector (Dr. Robert W. Sobol) (Vendetti et al. 2025). MEF WT and MEF UNG‐knockout cells were grown using D‐MEM, which included 10% heat‐inactivated FBS and 1% Pen/Strep.

#### 2.7.2 Treatment of Cell Lines

All treatments were performed with 5‐μM ceralasertib in both WT and UNG‐knockout (ΔUNG) cell lines. YUMM 1.7 cells were treated with ATRi for 30 min and 1 h. MEF cells were treated with ATRi for 4 and 8 h. B16F10 cells were treated with ATRi for 2 and 8 h. Following treatment, cells were collected for analysis using trypsin and pelleted for further processing.

#### 2.7.3 Purification of Genomic DNA

Genomic DNA was isolated and purified using the PureLink Genomic DNA Mini Kit (Invitrogen, K182002) according to manufacturer's instructions. Briefly, cell pellets were resuspended in PBS, and Proteinase K and RNase A were added. Following a brief incubation at room temperature, the lysis buffer was added and samples were incubated overnight at 55°C. Ethanol was added to the lysate which was then loaded onto a silica column and centrifuged at 13,000 RPM for 1 min. The column was washed twice with ethanol‐containing buffer, and genomic DNA was eluted with nuclease‐free water.

#### 2.7.4 Restriction Enzyme Digestion and Purification of DNA Fragments

Restriction enzyme digestion was performed using 5 μg of purified genomic DNA. Samples were treated with 20‐μg RNase A (Thermo Scientific, EN0531) and 5 units RNase H (New England BioLabs, M0297) in RNase H buffer (New England BioLabs, B0297) to remove contaminating RNA, followed by digestion with HindIII (Thermo Scientific, FD0504), EcoRI (Thermo Scientific, FD0274), BamHI (Fisher, FD0054) in FastDigest buffer (10X; Thermo Scientific, B64). Reactions were incubated overnight at 37°C. Nuclease‐free water was added to achieve the final reaction volume.

Purification of digested DNA fragments was done using the GeneJet PCR Purification Kit (Thermo Scientific, K0702) according to manufacturer's instructions. Briefly, binding buffer was added to digested DNA in a 1:1 volume ratio and samples were loaded onto the included silica‐based spin and centrifuged at 13,000 RPM for 1 min. The column was then washed, and DNA was eluted with nuclease‐free water.

#### 2.7.5 Digestion Into Nucleosides

**FIGURE 1:**
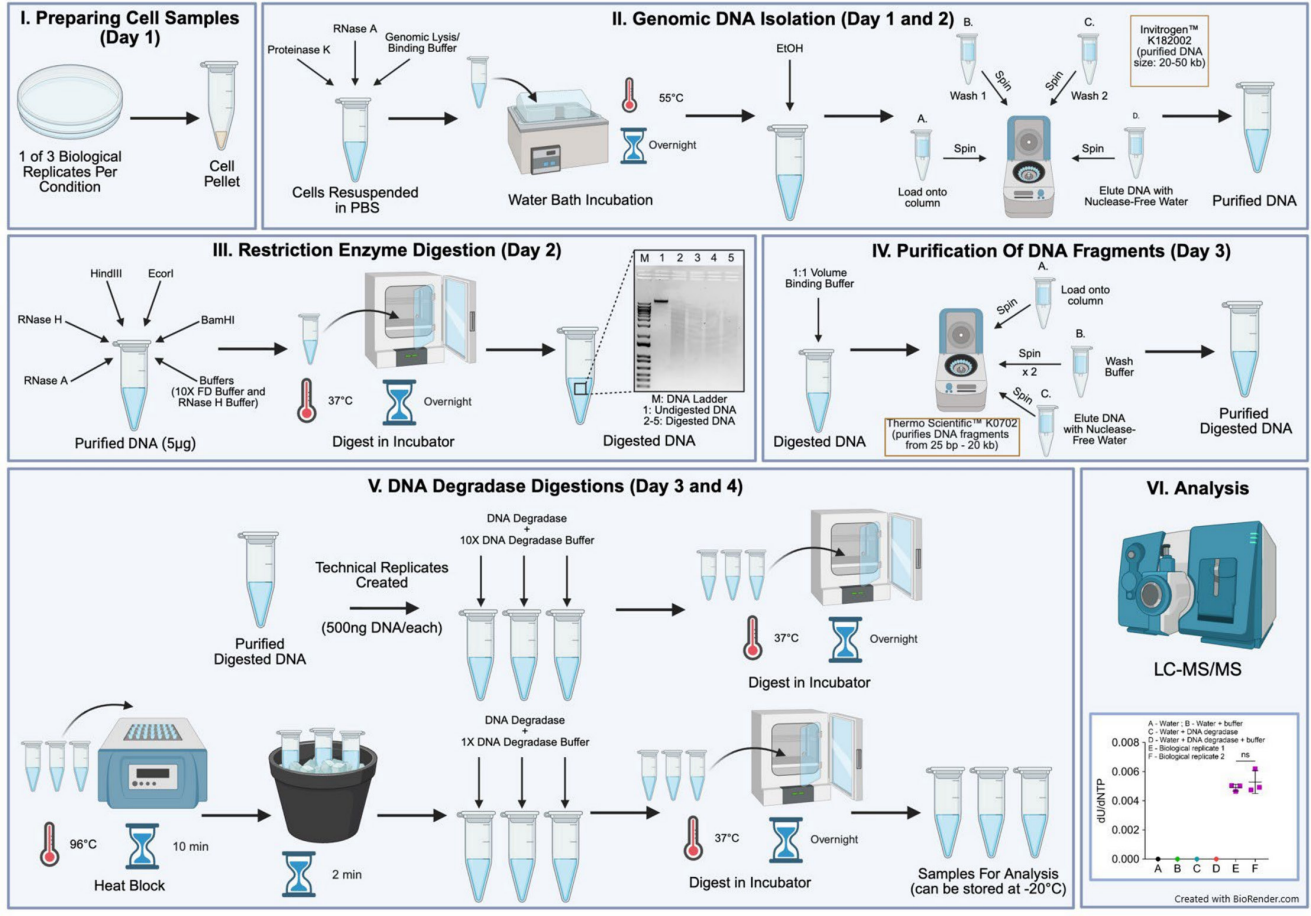
Method illustration for genomic DNA purification, digestion, and analysis.

Technical replicates were generated using 500 ng of DNA per sample. DNA was digested using DNA Degradase Plus (Zymo Research, E2021) in DNA Degradase buffer and incubated overnight at 37°C. The next day, replicates were heat‐inactivated at 96°C for 10 min and placed on ice for 2 min. A second digestion with DNA Degradase Plus (Zymo Research, E2021) with 1X DNA Degradase buffer was then performed overnight at 37°C to ensure complete hydrolysis of DNA into mononucleosides. Samples were stored at −80°C until analysis. Solvents used during DNA digestion were included in each LC–MS/MS run sequentially as follows: water alone, water:degradase (water and DNA Degradase Plus), water:buffer (water and DNA Degradase buffer), and water:degradase:buffer (water, DNA Degradase Plus, and DNA Degradase buffer). See Figure 1 for full illustration of genomic DNA purification and digestion. Statistical analyses were performed by ANOVA with Dunnett's multiple comparison test within each treatment cohort. Cells not treated with ATRi served as respective controls.

## 3 Results and Discussion

### 3.1 Validation of the Assay

#### 3.1.1 Chromatography

**FIGURE 2:**
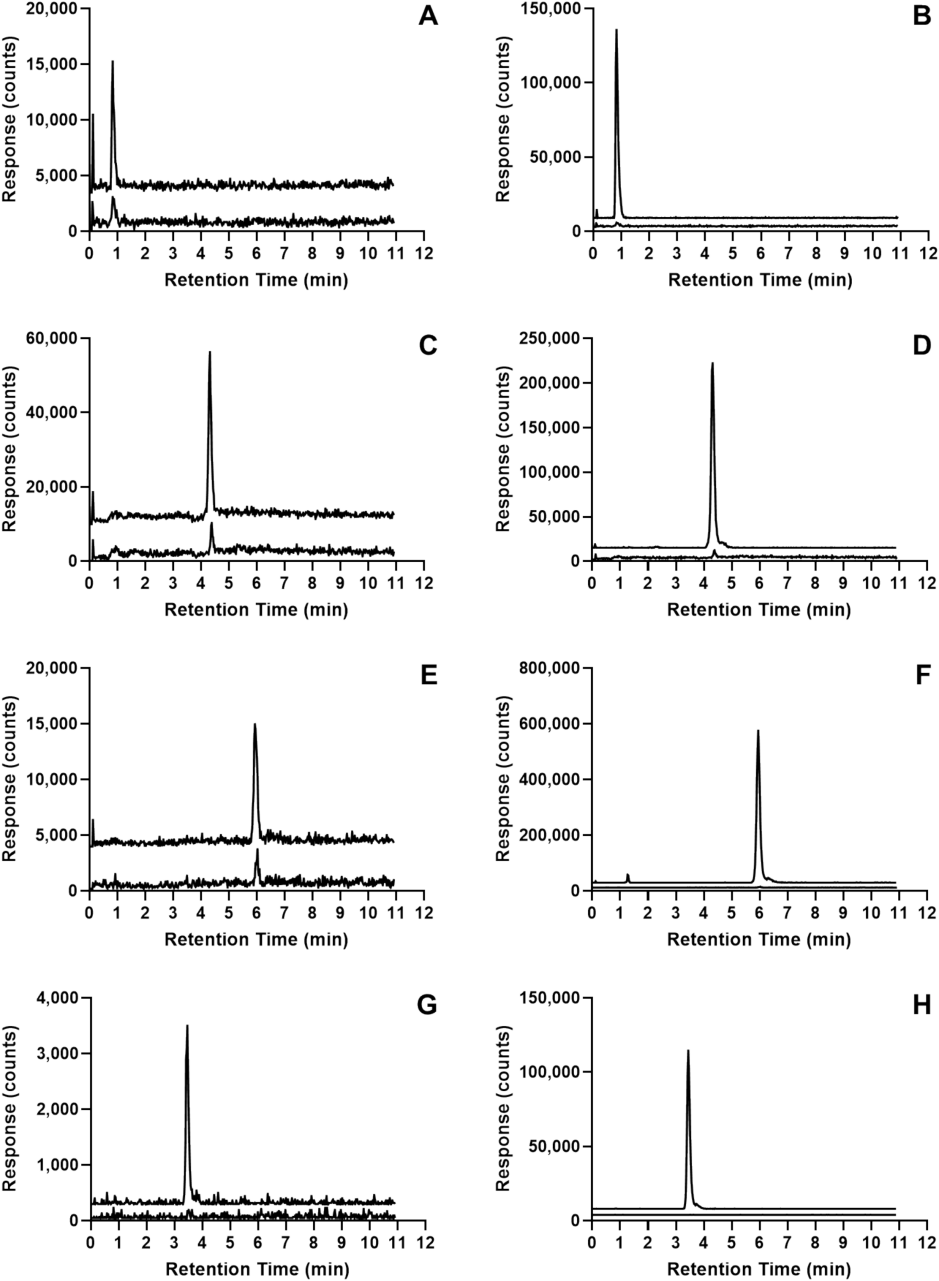
Representative chromatograms of rN analytes with corresponding internal standards. (A) Cyt (*m/z* 244.0 > 112.0) at LLOQ concentration (top trace with offset of 3500 counts) and blank control (bottom trace with offset of 200 counts); (B) Cyt‐IS (m/z 249.0 > 112.0) at concentration of 5 nM (top trace with offset of 9000 counts) and blank control (bottom trace with offset of 3000 counts); (C) Ade (*m/z* 268.0 > 136.0) at LLOQ concentration (top trace with offset of 10,000 counts) and blank control (bottom trace with offset of 20 counts); (D) Ade‐IS (*m/z* 273.0 > 136.0) at concentration of 5 nM (top trace with offset of 15,000 counts) and blank control (bottom trace with offset of 2000 counts); (E) Gua (*m/z* 284.1 > 152.1) at LLOQ concentration (top trace with offset of 30,000 counts) and blank control (bottom trace with offset of 200 counts); (F) Gua‐IS (*m/z* 283.0 > 162.0) at concentration of 5 nM (top trace with offset of 30,000 counts) and blank control (bottom trace with offset of 12,000 counts); (G) Urd (*m/z* 243.0 > 110.0) at LLOQ concentration (top trace with offset of 8000 counts) and blank control (bottom trace with 4000 counts); (H) Urd‐IS (*m/z* 246.0 > 111.0) at concentration of 100 nM (top trace with offset of 8000 counts) and blank control (bottom trace with offset of 4000 counts).

**FIGURE 3:**
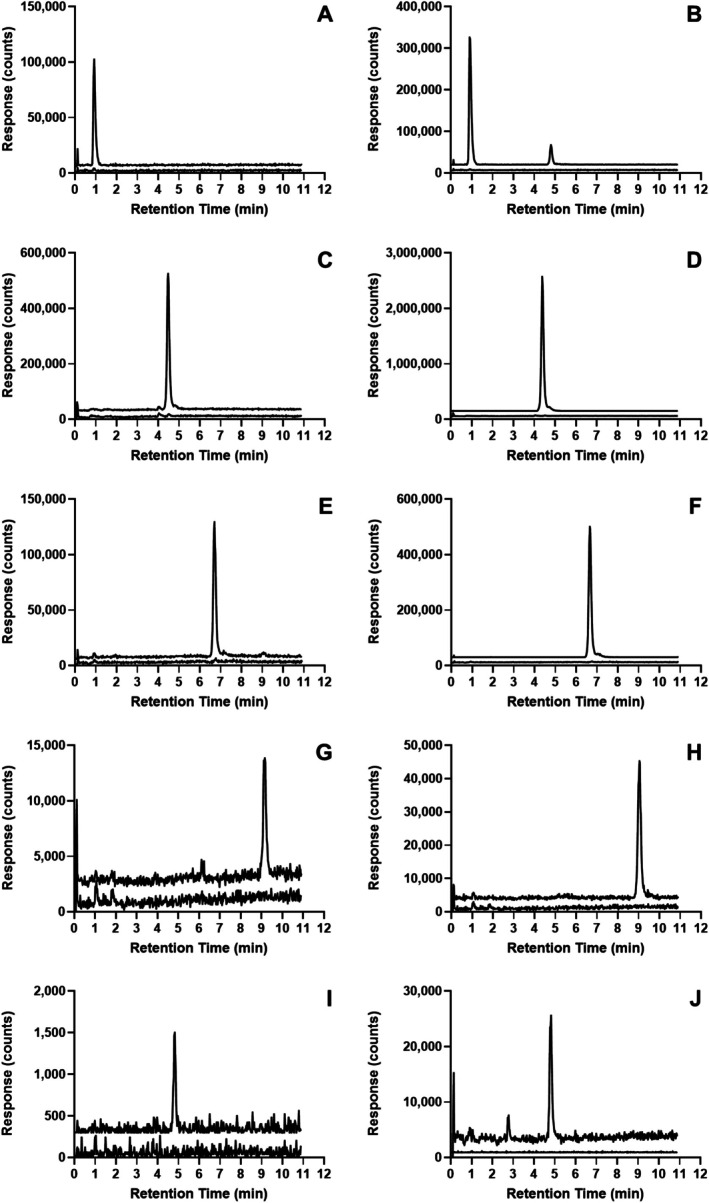
Representative chromatograms of dN analytes with corresponding internal standards: (A) dCyt (*m/z* 232.2 > 116.0) at LLOQ concentration (top trace with offset of 600 counts) and blank control (bottom trace with offset of 1000 counts); (B) dCyt‐IS (m/z 249.0 > 112.0) at concentration of 40 nM (top trace with offset of 20,000 counts) and blank control (bottom trace with offset of 6000 counts); (C) dAde (*m/z* 252.0 > 136.0) at LLOQ concentration (top trace with offset of 25,000 counts) and blank control (bottom trace with offset of 200 counts); (D) dAde‐IS (*m/z* 267.0 > 146.0) at concentration of 40 nM (top trace with offset of 150,000 counts) and blank control (bottom trace with offset of 50,000 counts); (E) dGua (*m/z* 268.0 > 152.0) at LLOQ concentration (top trace with offset of 6000 counts) and blank control (bottom trace with offset of 1000 counts); (F) dGua‐IS (*m/z* 268.0 > 152.0) at concentration of 40 nM (top trace with offset of 30,000 counts) and blank control (bottom trace with offset of 10,000 counts); (G) Thy (*m/z* 243.0 > 127.0) at LLOQ concentration (top trace with offset of 2000 counts) and blank control (bottom trace with 20 counts); (H) Thy‐IS (*m/z* 247.0 > 131.0) at concentration of 40 nM (top trace with offset of 3000 counts) and blank control (bottom trace with offset of 200 counts); (I) dUrd (*m/z* 227.0 > 184.0) at LLOQ concentration (top trace with offset of 300 counts) and blank control (bottom trace with 20 counts); (J) dUrd‐IS (*m/z* 230.0 > 185.0) at concentration of 100 nM (top trace with offset of 20 counts) and blank control (bottom trace with offset of 900 counts).

The approximate retention times for each compound were as follows: dUrd 4.9 min, Urd 3.5 min, Gua 6.0 min, Ade 4.3 min, Cyt 0.9 min, Thy 9.1 min, dGua 6.7 min, dAde 4.5 min, and dCyt 0.9 min. The analytes at the retention time extremes of approximately 0.9 and 9.1 min had corresponding capacity factors of 0.2 and 11.1, respectively. Void time was 0.75 min across analytes. Representative chromatograms of each rN and dN at the LLOQ and IS are displayed in Figure 2A–H and Figure 3A–J, respectively.

#### 3.1.2 Calibration Curve and LLOQ

The selected assay ranges of 1–100 nM (Gua, Ade, and Cyt), 10–1000 nM (Urd and dUrd), and 100–10,000 nM (Thy, dGua, dAde, and dCyt) fulfilled the FDA criteria for the LLOQ concentration and the calibration curve (U.S. Department of Health and Human Services Food and Drug Administration 2018). Analyte accuracies and precisions for each concentration were determined from triplicate calibration curves prepared on three separate days and are reported in Table S5. All analyte calibrator levels (minimum 6) were ±15% of nominal (theoretical) concentrations, meeting FDA requirements (U.S. Department of Health and Human Services Food and Drug Administration 2018). Representative calibration curves and corresponding correlation and regression coefficients are shown in Figure S1A–H for rNs and Figure S2A–J for dNs.

#### 3.1.3 Accuracy and Precision

All analyte intra‐ and interassay accuracies and precisions for the tested QC concentrations (LLOQ, QCL, QCM, and QCH) were within the defined acceptance criteria (accuracy: ±15% of nominal concentrations, except ±20% at LLOQ; precision: ≤ 15% CV, except ≤ 20% CV at LLOQ), as summarized in Table 1 (U.S. Department of Health and Human Services Food and Drug Administration 2018).

**TABLE 1:** Assay performance for the quantitation of LLOQ, QCL, QCM, and QCH for all analytes.

Analyte	Concentration (ng/mL)	Intra‐assay accuracy (%)	Interassay accuracy (%)	Intra‐assay precision (CV %)	Interassay precision (CV %)
Deoxyuridine	10 (LLOQ)	94.0–104.5	100.6	7.73	4.70
	15 (QCL)	101.4–111.8	108.2	5.35	4.97
	150 (QCM)	109.2–112.8	111.0	2.65	1.18
	800 (QCH)	99.6–105.3	102.6	4.52	2.09
Uridine	10 (LLOQ)	89.1–101.4	96.6	6.17	6.33
	15 (QCL)	110.0–113.2	111.3	3.26	0.079
	150 (QCM)	111.6–112.0	111.9	3.18	*
	800 (QCH)	94.6–97.9	95.8	3.08	1.41
Guanosine	1.0 (LLOQ)	88.8–99.7	94.5	6.11	5.21
	1.5 (QCL)	108.1–109.1	108.7	7.61	*
	15.0 (QCM)	104.9–109.7	107.2	3.49	1.72
	80.0 (QCH)	90.1–91.4	90.8	3.23	*
Adenosine	1.0 (LLOQ)	92.3–98.0	95.1	6.08	1.72
	1.5 (QCL)	112.6–113.6	113.2	2.90	*
	15.0 (QCM)	113.9–114.1	114.2	2.20	0.25
	80.0 (QCH)	102.7–107.7	104.6	3.97	2.02
Cytidine	1.0 (LLOQ)	88.3–101.2	94.3	7.03	6.27
	1.5 (QCL)	103.9–109.8	106.2	3.98	2.46
	15.0 (QCM)	110.0–111.6	111.0	2.09	*
	80.0 (QCH)	95.4–99.4	96.9	2.39	2.09
Thymidine	100 (LLOQ)	89.0–101.2	96.7	6.00	6.43
	150 (QCL)	108.6–111.3	110.2	3.15	0.36
	1500 (QCM)	106.2–109.9	107.5	3.90	1.11
	8000 (QCH)	92.4–99.5	95.9	3.49	3.40
Deoxyguanosine	100 (LLOQ)	99.0–107.5	101.9	3.53	4.53
	150 (QCL)	112.0–114.2	111.7	2.32	2.20
	1500 (QCM)	107.3–109.4	109.3	3.16	1.08
	8000 (QCH)	89.9–98.3	94.6	2.51	4.45
Deoxyadenosine	100 (LLOQ)	96.1–103.1	99.8	3.74	3.19
	150 (QCL)	110.1–111.4	110.9	2.72	*
	1500 (QCM)	110.1–112.0	110.8	2.19	0.34
	8000 (QCH)	91.4–96.5	94.3	3.48	2.42
Deoxycytidine	100 (LLOQ)	95.0–102.3	99.6	2.78	3.81
	150 (QCL)	110.6–112.1	111.4	2.72	*
	1500 (QCM)	109.7–112.6	111.0	2.18	0.98
	8000 (QCH)	93.9–97.6	96.3	1.57	2.04

*Note: N* = 18/analyte; sixfold results, each in three separate runs for each concentration. Asterisks denote that the mean square of the within runs was greater than the mean square of the between runs, indicating that there was no significant additional variation due to the performance of the assay in different runs (Rosing et al. 2000).

Abbreviations: LLOQ, lower limit of quantitation; QCL, QC low; QCM, QC mid; QCH, QC high.

#### 3.1.4 Selectivity and Specificity

The mean cross talk of all analytes into respective IS channels was 0.01% (range: 0.0004% to 0.03%). The mean cross talk of IS into respective analyte channels was 0.14% (range: 0.01% to 0.44%) (Table S6).

Average carryover across analytes was 0.54% of the original respective LLOQ (range: 0.31% to 0.85%) in sequential blank injections (Table S7 and Figure S3A–I). Defining the maximum carryover as 20% of the LLOQ area, this would mean that only samples containing a dN concentration of more than approximately 18,518 nM (tenfold lower for dUrd/Urd; 100‐fold lower for Gua, Ade, and Cyt) could result in unacceptable carryover into the next sample.

#### 3.1.5 Stability

Across individual analytes, the stability of analyte stock solution ranged from 90.8% to 98.4% at ambient temperature for 6 h and 88.9% to 104.0% after 2 years at −80°C. Analyte‐specific stock and QC stabilities are summarized in Tables S8–S16. Benchtop stability of QCs after four at ambient temperature with ambient light ranged between 104.1% and 114.0%. The stability of the analytes after 3 freeze–thaw cycles (−80°C to ambient temperature) were between 106.4% and 114.3%. Long‐term QC stability after 12 months at −80°C was adequate with recovery between 88.7% and 113.7%. Accuracy of back‐calculated QC concentrations of all analytes when kept in the autosampler for 72 h ranged from 94.2% to 110.3% of the initial response, fulfilling the requirements set by the FDA (U.S. Department of Health and Human Services Food and Drug Administration 2018).

#### 3.1.6 Dilutional Integrity

Dilutional integrity assessment was evaluated for all analytes. The accuracies were as follows: 109.3% (CV 2.3%) for dUrd, 112.0% (CV 3.2%) for Urd, 109.0% (CV 3.3%) for Gua, 112.7% (CV 2.7%) for Ade, 110.7% (CV 1.4%) for Cyt, 104.3% (CV 2.0%) for Thy, 110.0% (CV 3.1%) for dGua, 106.3% (CV 5.3%) for dAde, and 109.7% (CV 1.9%) for dCyt.

### 3.2 Development

The method development of the assay included multiple variations in column type, UPLC gradients, buffers, and approaches aimed to optimize chromatography and to minimize carryover and background contamination. Initially, we applied a method developed by Tsuji et al., which measured modified and canonical DNA nucleosides, as a framework that was then optimized to measure our analytes of interest (Tsuji et al. 2014). Development began with MS tuning to optimize parameters of each analyte and to obtain sensitive and specific detection. Each analyte was scanned in both ESI negative and positive ionization modes until the most sensitive mode was identified on an analyte‐specific basis. We determined that dUrd and Urd displayed higher sensitivity in negative ionization, whereas the other analytes had higher sensitivity in positive ionization mode. Because we expected high concentrations of dNs in digested DNA specimens, an anticipated problem was the potential of MS detector saturation. Therefore, isotopologue‐MRM channels were implemented for these analytes in a similar manner as previously described (Parise et al. 2021). However, response remained linear with all primary MRM pairs and isotopologue‐MRM channels were ultimately not needed. Furthermore, sensitivity of the assay was not compromised and was proven to be sufficient for the intended application (see below).

We evaluated the following three columns: Acquity BEH UPLC HILIC (100 × 2.1 mm, 1.7 μm), Inertsil ODS‐3 (150 × 2.1 mm, 2 μm), and Inertsil ODS‐3 (150 × 2.1 mm, 3 μm). The BEH (HILIC) column showed extreme tailing of some analytes that resulted in inadequate peak widths of greater than 4 min and was therefore not chosen for further development. The two Inertsil ODS‐3 columns allowed for adequate separation of the nine analytes. However, the larger particle size column (3 μm) allowed for lower backpressure and was therefore selected for further development. The gradient program was determined empirically by changing the percentage of aqueous and organic and flow over time to optimize resolution and analyte separation (See Section 2.2 for details). Initial experiments tested acetonitrile as the organic mobile phase solvent; however insufficient retention of the first eluting compound (dCyt) was observed, and methanol was subsequently chosen to achieve adequate resolution. Ammonium formate was also evaluated as a volatile mobile phase additive to help improve ionization and peak shape but provided no additional benefit compared to methanol alone. Test injections began at 5 μL, which resulted in insufficient signal‐to‐noise analyte peaks. Subsequent 10‐μL injections were tested and resulted in some improvement but failed to provide reliable detection in our LLOQ calibrators. Finally, a 20‐μL injection resulted in reliable low‐concentration detection and adequate signal‐to‐noise ratios across all analytes. IS concentrations were chosen such that peak area was comparable to the peak area of their respective calibrators in the middle of the calibration curve. The use of a column heater set at 40°C resulted in narrower and more uniform chromatographic peaks.

A dilute‐and‐shoot approach was determined to be the most reasonable and appropriate sample preparation as cell digests were prepared in water with no complex matrix (e.g., plasma or tissue homogenate) that required extraction methods such as protein precipitation, liquid–liquid extraction, or solid‐phase extraction. These extra extraction methods could also unnecessarily result in analyte loss, variability, false positive contamination with nucleosides, and/or add unneeded processing time. We therefore did not evaluate alternative preparation strategies during our preliminary studies. Because cell digestions were already prepared in water and did not require cleanup, the 1:2 sample‐to‐internal‐standard dilution ratio allowed for a fixed dilution volume to be applied across standards, QCs, and unknown samples while also providing robust IS signal and provided sufficient final sample volume needed for injection.

Given the extreme sensitivity of our method's detection, particularly for rN analytes, a central focus of this method's development was the rigorous control of digestion reagent contamination during sample processing. Multiple degradase enzymes, buffer systems, and solvents (e.g., water) were systemically evaluated under controlled conditions, including the testing of different vendors, to identify and limit exogenous sources of rNs. Concurrent controls, such as buffer‐ and degradase‐only preparations, were included alongside experimental samples to actively monitor for any reagent‐derived contamination and/or nonspecific background signals. Therefore, any detected analyte signal could be attributed to the associated sample and distinguished from signal introduced during DNA digestion or sample preparation, thereby strengthening the rigor, specificity, and interpretability of our measurements.

With the chosen ULOQ for dNs (10,000 nM) and LLOQ for rNs (1 nM) and dUrd/Urd (10 nM), this method would theoretically be capable of detecting ribose and uracil nucleoside misincorporation events down to a ratio of 1 in 10,000 (0.01%) or 1 in 1000 (0.1%), respectively. As elevated ribonucleotide incorporation is associated with genomic instability and may lead to cancer (Potenski and Klein 2014), even small increases in misincorporation can influence drug sensitivity, mutation signatures, and tumor evolution.

Beyond the validation and application experiments described here, our method has been applied as a fit‐for‐purpose assay for quantitative analysis of samples across multiple investigational studies over 4 years (Pandya et al. 2024; Sugitani et al. 2022). Continued utility over time shows our method has been reproducible and practically useful in real study workflows. Alternative nucleoside analogs, such as 5‐methyl‐2′‐deoxycytidine (5mdC), 5‐hydroxymethyl‐2′‐deoxycytidine (5hmdC), 5‐bromo‐2′‐deoxyuridine (BrdU), and 5‐chloro‐2′‐deoxyrudine (CldU) may also be added to our method for potential quantitative evaluation of cell proliferation, gene regulation, and/or treatment‐related effects on DNA metabolism.

### 3.3 Assay Application

#### 3.3.1 Baseline Levels of rNs and dNs in Genomic DNA Across Cell Lines

Each tested cell line provided quantitative data of rNs and dNs. No contamination was found in water or buffer; however, control samples of DNA degradase were found to be contaminated with Urd, Gua, Ade, and Cyt at concentrations similar to what was observed in our treatment samples. Degradase‐induced contamination of Thy, dGua, dAde, and dCyt was also observed, albeit to a much lesser extent, and ultimately determined to not be a significant contributing source of dNs in any of our treatment samples. No contamination of dUrd was observed in any reagents. Thus, a dUrd/dN ratio (i.e., dUrd/[dAde+dCyt+dGua+Thy]) was designated as the primary outcome for subsequent analyses across all cell lines. Representative rN and dN concentrations generated from treated cells can be seen in Table S17.

#### 3.3.2 YUMM 1.7 Cells

**FIGURE 4:**
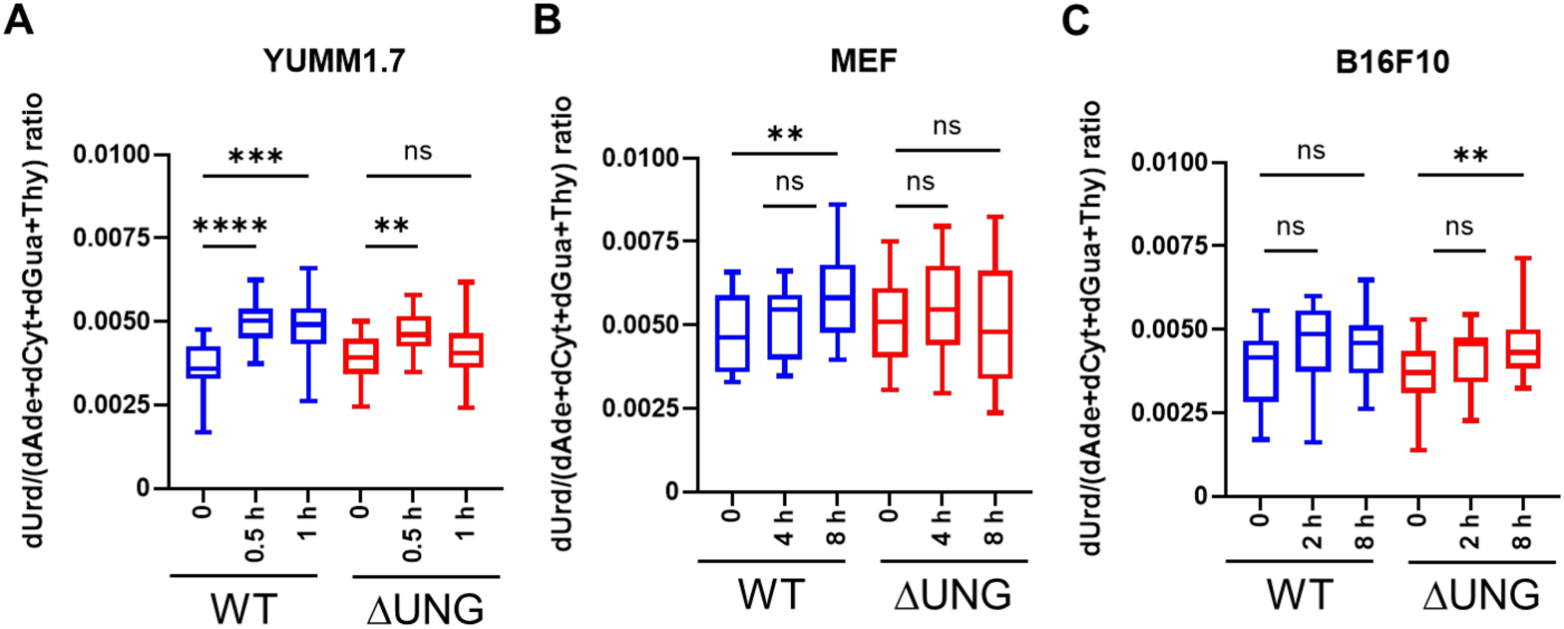
dUrd/(dAde+dCyt+dGua+Thy) ratios across different cell lines over time following 5‐μM ATRi treatment. (A) WT and ΔUNG YUMM1.7, (B) WT and ΔUNG MEF, and (C) WT and ΔUNG B16F10. The data reflect experimental replicates with each condition containing technical triplicates per experiment. Box and whiskers plot show the interquartile range, spanning the middle 50% of the data with extensions to lowest and highest values. Statistical comparison was conducted by ANOVA with Dunnett's multiple comparison test within each treatment cohort. Untreated (0 h) samples served as respective controls. **p < 0.01, ***p < 0.001, and ****p < 0.0001.

In the WT YUMM 1.7 cell line, ATRi‐treated cells demonstrated a significant increase in the dUrd/dN ratio at 0.5 h (*p* < 0.0001; ~41% increase) and 1 h (*p* < 0.001; ~30% increase) compared to untreated controls. Among YUMM 1.7 ΔUNG cells, treatment with ATRi for 0.5 h demonstrated a significant increase (*p* < 0.01) in the dUrd/dN ratio by approximately 19% compared to untreated controls. Therefore, WT YUMM 1.7 cells treated with ATRi were associated with more prolonged dUrd contamination compared with YUMM 1.7 ΔUNG cells (Figure 4A).

#### 3.3.3 MEF Cells

The dUrd/dN ratio in MEF WT cells significantly increased in cells treated with ATRi for 8 h compared to untreated controls (*p* < 0.01; ~23% increase). The dUrd/dN ratio was not significantly different in MEF ΔUNG cells with any treatment conditions. Thus, treating MEF WT cells with ATRi induced greater dUrd contamination compared with ATRi‐treated MEF ΔUNG cells (Figure 4B).

#### 3.3.4 B16F10 Cells

Among WT B16F10 cells treated with 5‐μM ATRi, the dUrd/dN ratio was not significantly different at 2 h (*p* = 0.188) or 8 h (*p* = 0.160) compared to untreated controls. However, a significant difference (*p* < 0.01) in the dUrd/dN ratio occurred in B16F10 ΔUNG cells at 8 h posttreatment with ATRi and demonstrated an increase of approximately 22% when compared to untreated controls (Figure 4C).

## 4 Conclusion

The objective of this study was to develop an LC–MS/MS method for the quantification of rNs and dNs in digested genomic DNA. The assay proved to be accurate, precise, reliable, and applicable across multiple cell lines treated with ceralasertib. Utility of our method provided evidence that ceralasertib‐induced ATR inhibition induces dU contamination in WT B16F10, YUMM 1.7, and MEF cell lines. This bioanalytical method represents a critical tool for understanding the impact of alterations to rN and dN pools, particularly when pharmacologically inhibiting ATR.

## Conflicts of Interest

J.H.B. reports spouse salary from AstraZeneca.

## Supporting information


**Figure S1:** Representative calibration curves and residuals (*N* = 3/level) for rN analytes. (A,B) Cyt (response = 0.0747 • conc +0.0196; *R* ^2^ = 0.998); (C,D) Ade (response = 0.1520 • conc +0.0440; *R* ^2^ = 0.999); (E,F) Gua (response = 0.0156 • conc +0.0037; *R* ^2^ = 0.996); (G,H) Urd (response = 0.0027 • conc—0.0003; *R* ^2^ = 0.998). Calibration curves are depicted as response ratio versus nominal concentration and associated residuals (%) are representative of the back‐calculated relative to the nominal concentrations versus the log‐transformed concentration.
**Figure S2:** Representative calibration curves and residuals (*N* = 3/level) for dN analytes. (A,B) dUrd (response = 0.0041 • conc—0.0013; *R* ^2^ = 0.998); (C,D) Thy (response = 0.0024 • conc +0.0190; *R* ^2^ = 0.998); (E,F) dGua (response = 0.0025 • conc—0.0034; *R* ^2^ = 0.998); (G,H) dAde (response = 0.0020 • conc +0.0024; *R* ^2^ = 0.999); (I,J) dCyt (response = 0.0029 • conc +0.0092; *R* ^2^ = 0.999). Calibration curves are depicted as response ratio versus nominal concentration and associated residuals (%) are representative of the back‐calculated relative to the nominal concentrations versus the log‐transformed concentration.
**Figure S3:** Analytical carryover of: (A) Cyt (B) Ade (C) Gua (D) Urd (E) dUrd (F) Thy (G) dGua (H) dAde (I) dCyt described in serial blank injections following a spiked analyte sample 10× ULOQ. Data represented as mean percent analyte response relative to 10× ULOQ response ± SD from three independent runs.
**Table S1:** Mass spectrometric conditions for ESI negative analytes and internal standards.
**Table S2:** Mass spectrometric conditions for ESI positive analytes and internal standards.
**Table S3:** Calibration curve levels with corresponding concentrations.
**Table S4:** Quality control concentrations.
**Table S5:** Assay performance of calibrators.
**Table S6:** Cross talk for all analytes.
**Table S7:** Carryover performance.
**Table S8:** Stability of deoxyuridine under varying conditions.
**Table S9:** Stability of uridine under varying conditions.
**Table S10:** Stability of guanosine under varying conditions.
**Table S11:** Stability of adenosine under varying conditions.
**Table S12:** Stability of cytidine under varying conditions.
**Table S13:** Stability of thymidine under varying conditions.
**Table S14:** Stability of deoxyguanosine under varying conditions.
**Table S15:** Stability of deoxyadenosine under varying conditions.
**Table S16:** Stability of deoxycytidine under varying conditions.
**Table S17:** Representative rN and dN concentrations from WT and ΔUNG MEF cells exposed to 5‐μM ceralasertib for 0, 4, and 8 h.
