# Supplementary material for: Resolution and Quantitation of Ribonucleosides and Deoxyribonucleosides in Digested Genomic DNA by UHPLC–MS/MS
Source: Biomed Chromatogr. 2026 Aug 13;40(10):e70599. doi: 10.1002/bmc.70599 (PMC13473814; doi:10.1002/bmc.70599)
Supplement: Supplementary file 1 [file BMC-40-e70599-s001.docx]

# Supplementary File

Resolution and quantitation of ribonucleosides and deoxyribonucleosides in digested genomic DNA by UHPLC-MS/MS

Joshua J. Deppas^1,2*^, Reyna Jones^3*^, Robert A. Parise^2^, Pinakin Pandya^1^, Christopher J. Bakkenist^1,4^, Jan H. Beumer^2,3,5^

*equal contributors

^1^Cancer Therapeutics Program, University of Pittsburgh Cancer Institute, Pittsburgh, PA

^2^Department of Pharmaceutical Sciences, School of Pharmacy, University of Pittsburgh, Pittsburgh, PA

^3^Department of Radiation Oncology, School of Medicine, University of Pittsburgh, Pittsburgh, PA, USA.

^4^Department of Pharmacology and Chemical Biology, School of Medicine, University of Pittsburgh, Pittsburgh, Pennsylvania, PA

^5^ Department of Oncology, Johns Hopkins University School of Medicine and Johns Hopkins Sidney Kimmel Cancer Center

Emails: [jod102@pitt.edu](mailto:jod102@pitt.edu); [rej27@pitt.edu](mailto:rej27@pitt.edu); [parisera@upmc.edu](mailto:parisera@upmc.edu); [pip10@pitt.edu](mailto:pip10@pitt.edu); [cjb38@pitt.edu](mailto:cjb38@pitt.edu); [jan.beumer@jhu.edu](mailto:jan.beumer@jhu.edu)

Correspondence:

Jan H. Beumer

Department of Oncology, Johns Hopkins University School of Medicine and Johns Hopkins Sidney Kimmel Cancer Center

1650 Orleans St, Room 1M52, Baltimore, MD 21287, USA

Tel.: +1-443-287-6476

Email: beumerj@gmail.com

Key words: ribonucleosides, deoxynucleosides, nucleoside quantitation, LC-MS/MS, DNA assay, ATR inhibitor, ceralasertib

# Supplementary Tables

Suppl.Table 1. Mass spectrometric conditions for ESI negative analytes and internal standards

| **Parameter** | **dUrd** | **dUrd-IS** | **Urd** | **Urd-IS** |
| --- | --- | --- | --- | --- |
| ESI | Neg | Neg | Neg | Neg |
| CG | 20 | 20 | 20 | 20 |
| CAD | 7 | 7 | 7 | 7 |
| IS Voltage (V) | - 4,500 | - 4,500 | - 4,500 | - 4,500 |
| TEM (°C) | 300 | 300 | 300 | 300 |
| GS1 | 70 | 70 | 70 | 70 |
| GS2 | 70 | 70 | 70 | 70 |
| EP (V) | -10 | -10 | -10 | -10 |
| CXP | -15 | -15 | -15 | -15 |
| DP (V) | -40 | -40 | -40 | -40 |
| CE (V) | -20 | -20 | -20 | -20 |
| DT (msec) | 50 | 50 | 50 | 50 |
| Q1 *m/z* | 227.0 | 230.0 | 243.0 | 246.0 |
| Q3 *m/z* | 184.0 | 185.0 | 110.0 | 111.0 |
| Expected RT (min) | 4.9 | 4.8 | 3.5 | 3.4 |

Suppl.Table 2. Mass spectrometric conditions for ESI positive analytes and internal standards

| **Parameter** | **Gua** | **Gua-IS** | **Ade** | **Ade-IS** | **Cyt** | **Cyt-IS** | **Thy** | **Thy-IS** | **dGua** | **dGua-IS** | **dAde** | **dAde-IS** | **dCyt** | **dCyt-IS** |
| --- | --- | --- | --- | --- | --- | --- | --- | --- | --- | --- | --- | --- | --- | --- |
| ESI | Pos | Pos | Pos | Pos | Pos | Pos | Pos | Pos | Pos | Pos | Pos | Pos | Pos | Pos |
| CG | 20 | 20 | 20 | 20 | 20 | 20 | 20 | 20 | 20 | 20 | 20 | 20 | 20 | 20 |
| CAD | 7 | 7 | 7 | 7 | 7 | 7 | 7 | 7 | 7 | 7 | 7 | 7 | 7 | 7 |
| IS Voltage (V) | 4,500 | 4,500 | 4,500 | 4,500 | 4,500 | 4,500 | 4,500 | 4,500 | 4,500 | 4,500 | 4,500 | 4,500 | 4,500 | 4,500 |
| TEM (°C) | 300 | 300 | 300 | 300 | 300 | 300 | 300 | 300 | 300 | 300 | 300 | 300 | 300 | 300 |
| GS1 | 70 | 70 | 70 | 70 | 70 | 70 | 70 | 70 | 70 | 70 | 70 | 70 | 70 | 70 |
| GS2 | 70 | 70 | 70 | 70 | 70 | 70 | 70 | 70 | 70 | 70 | 70 | 70 | 70 | 70 |
| EP (V) | 10 | 10 | 10 | 10 | 10 | 10 | 10 | 10 | 10 | 10 | 10 | 10 | 10 | 10 |
| CXP | 10 | 10 | 10 | 10 | 10 | 10 | 10 | 10 | 10 | 10 | 10 | 10 | 10 | 10 |
| DP (V) | 30 | 30 | 20 | 20 | 20 | 20 | 30 | 30 | 20 | 20 | 20 | 20 | 20 | 20 |
| CE (V) | 15 | 15 | 20 | 20 | 20 | 20 | 20 | 20 | 20 | 20 | 20 | 20 | 15 | 15 |
| DT (msec) | 50 | 50 | 50 | 50 | 50 | 50 | 50 | 50 | 50 | 50 | 50 | 50 | 50 | 50 |
| Q1 *m/z* | 284.1 | 287.0 | 268.0 | 273.0 | 244.0 | 249.0 | 243.0 | 247.0 | 268.0 | 283.0 | 252.0 | 267.0 | 228.0 | 232.2 |
| Q3 *m/z* | 152.1 | 155.0 | 136.0 | 136.0 | 112.0 | 112.0 | 127.0 | 131.0 | 152.0 | 162.0 | 136.0 | 146.0 | 112.0 | 116.0 |
| Expected RT (min) | 6.0 | 5.9 | 4.3 | 4.3 | 0.9 | 0.8 | 9.1 | 9.0 | 6.7 | 6.6 | 4.5 | 4.4 | 0.9 | 0.9 |

Suppl.Table 3. Calibration curve levels with corresponding concentrations

| **Calibrator**  **Level** | **Thy, dGua, dAde, and dCyt**  **Conc. (nM)** | **dUrd and Urd**  **Conc. (nM)** | **Gua, Ade, and Cyt**  **Conc. (nM)** |
| --- | --- | --- | --- |
| Level 7 | 10,000 | 1,000 | 100 |
| Level 6 | 5,000 | 500 | 50 |
| Level 5 | 2,000 | 200 | 20 |
| Level 4 | 1,000 | 100 | 10 |
| Level 3 | 500 | 50 | 5 |
| Level 2 | 200 | 20 | 2 |
| Level 1 | 100 | 10 | 1 |
| Level 0 | 0 | 0 | 0 |

Suppl.Table 4. Quality control concentrations

| **Level** | **Thy, dGua, dAde, and dCyt**  **Conc. (nM)** | **dUrd and Urd**  **Conc. (nM)** | **Gua, Ade, and Cyt**  **Conc. (nM)** |
| --- | --- | --- | --- |
| QCH | 8,000 | 800 | 80 |
| QCM | 1,500 | 150 | 15 |
| QCL | 150 | 15 | 1.5 |
| LLOQ | 100 | 10 | 1.0 |

QCH, QC High; QCM, QC Mid; QCL, QC Low; LLOQ, Lower limit of quantitation

Suppl.Table 5. Assay performance of calibrators

| **Analyte** | **Concentration**  **(nM)** | **Intra-assay Accuracy (%)** | **Inter-assay Accuracy (%)** | **Intra-assay precision (%)** | **Inter-assay precision (%)** |
| --- | --- | --- | --- | --- | --- |
| Deoxyuridine | 10 | 99.2-100.7 | 99.6 | 8.00 | * |
|  | 20 | 100.5-103.5 | 101.9 | 5.74 | * |
|  | 50 | 102.3-103.6 | 102.9 | 5.05 | * |
|  | 100 | 98.3-102.3 | 100.6 | 5.56 | * |
|  | 200 | 96.0-102.7 | 98.5 | 9.11 | * |
|  | 500 | 99.7-103.8 | 103.5 | 7.71 | * |
|  | 1,000 | 99.2-98.7 | 98.1 | 4.76 | * |
| Uridine | 10 | 99.4-101.1 | 100.1 | 6.445 | * |
|  | 20 | 97.8-100.5 | 100.6 | 3.11 | 1.19 |
|  | 50 | 100.7-102.4 | 101.4 | 2.35 | * |
|  | 100 | 99.9-102.9 | 101.8 | 4.69 | * |
|  | 200 | 96.2-101.0 | 99.0 | 3.16 | 1.02 |
|  | 500 | 98.1-107.8 | 101.8 | 4.76 | 2.52 |
|  | 1,000 | 95.6-99.4 | 97.4 | 1.72 | 0.99 |
| Guanosine | 1.0 | 98.7-101.6 | 100.6 | 8.89 | * |
|  | 2.0 | 98.2-100.3 | 99.8 | 6.72 | * |
|  | 5.0 | 100.1-101.0 | 103.4 | 4.58 | 0.89 |
|  | 10.0 | 100.1-106.5 | 100.9 | 3.36 | 2.24 |
|  | 20.0 | 99.2-101.8 | 100.2 | 5.06 | * |
|  | 50.0 | 96.8-108.4 | 101.4 | 6.69 | 2.70 |
|  | 100.0 | 95.3-97.7 | 96.8 | 3.38 | * |
| Adenosine | 1.0 | 98.6-101.3 | 99.8 | 0.01 | 1.06 |
|  | 2.0 | 97.3-103.7 | 100.3 | 2.23 | 1.66 |
|  | 5.0 | 99.4-101.3 | 100.3 | 2.27 | * |
|  | 10.0 | 98.7-103.5 | 101.8 | 4.58 | 0.01 |
|  | 20.0 | 98.8-103.2 | 100.4 | 4.42 | * |
|  | 50.0 | 100.5-102.1 | 101.1 | 5.52 | * |
|  | 100.0 | 94.8-99.7 | 97.6 | 2.58 | 1.20 |
| Cytidine | 1.0 | 98.8-101.5 | 100.1 | 7.07 | * |
|  | 2.0 | 97.8-102.2 | 99.4 | 4.21 | 0.01 |
|  | 5.0 | 102.5-103.9 | 103.2 | 2.53 | * |
|  | 10.0 | 97.9-104.8 | 101.6 | 4.67 | 1.20 |
|  | 20.0 | 97.0-99.5 | 98.3 | 4.29 | * |
|  | 50.0 | 97.7-108.7 | 102.0 | 3.77 | 3.07 |
|  | 100.0 | 97.2-99.3 | 98.1 | 2.83 | * |
| Thymidine | 100 | 99.6-102.7 | 100.8 | 7.13 | * |
|  | 200 | 95.7-102.3 | 98.9 | 3.55 | 1.54 |
|  | 500 | 100.6-106.5 | 103.0 | 4.06 | 1.08 |
|  | 1,000 | 99.3-102.7 | 101.3 | 6.20 | * |
|  | 2,000 | 95.5-104.5 | 99.9 | 3.34 | 2.35 |
|  | 5,000 | 95.3-107.7 | 101.4 | 4.30 | 3.23 |
|  | 10,000 | 96.7-99.3 | 98.2 | 3.07 | * |
| Deoxyguanosine | 100 | 98.0-101.2 | 99.9 | 2.82 | 0.33 |
|  | 200 | 95.8-101.5 | 98.8 | 2.65 | 1.41 |
|  | 500 | 99.9-107.1 | 103.5 | 3.52 | 1.61 |
|  | 1,000 | 98.4-104.8 | 102.1 | 4.82 | 0.99 |
|  | 2,000 | 97.5-100.3 | 98.8 | 4.57 | * |
|  | 5,000 | 97.3-110.1 | 102.3 | 4.95 | 3.49 |
|  | 10,000 | 95.8-97.9 | 97.1 | 2.93 | * |
| Deoxyadenosine | 100 | 97.8-100.6 | 99.5 | 2.73 | * |
|  | 200 | 97.5-101.0 | 99.2 | 2.06 | 0.75 |
|  | 500 | 102.5-107.1 | 104.4 | 2.42 | 1.07 |
|  | 1,000 | 99.6-104.3 | 102.7 | 4.50 | 0.37 |
|  | 2,000 | 97.5-101.2 | 99.8 | 3.00 | 0.57 |
|  | 5,000 | 97.3-103.4 | 99.9 | 3.96 | 1.25 |
|  | 10,000 | 94.0-98.7 | 95.8 | 3.46 | 0.98 |
| Deoxycytidine | 100 | 98.0-100.3 | 99.5 | 2.10 | 0.32 |
|  | 200 | 98.7-101.3 | 99.9 | 2.02 | 0.38 |
|  | 500 | 101.2-107.5 | 103.4 | 1.48 | 1.92 |
|  | 1,000 | 97.1-103.8 | 100.4 | 3.96 | 1.42 |
|  | 2,000 | 95.8-101.0 | 99.0 | 2.57 | 1.37 |
|  | 5,000 | 97.7-108.3 | 101.7 | 4.31 | 2.96 |
|  | 10,000 | 96.5-99.5 | 98.0 | 1.68 | 0.55 |

Data represents 3 analytical runs. Asterisks denote that the mean square of the within runs was greater than the mean square of the between runs, indicating that there was no significant additional variation due to the performance of the assay in different runs [16].

Suppl.Table 6. Cross-talk for all analytes

| **Analyte** | **10x ULOQ**  **(nM)** | **Mean Cross-Talk of IS into Analyte Channel (%)** | **Mean Cross-Talk of Analyte into IS Channel (%)** |
| --- | --- | --- | --- |
| Thymidine | 100,000 | 4.07E-03 | 3.85E-01 |
| Deoxyguanosine | 100,000 | 6.64E-04 | 8.50E-03 |
| Deoxyadenosine | 100,000 | 3.77E-04 | 9.59E-02 |
| Deoxycytidine | 100,000 | 3.33E-03 | 6.08E-03 |
| Deoxyuridine | 10,000 | 2.85E-02 | 3.16E-02 |
| Uridine | 10,000 | 3.30E-02 | 4.15E-02 |
| Guanosine | 1,000 | 8.00E-03 | 1.26E-01 |
| Adenosine | 1,000 | 1.67E-03 | 4.36E-01 |
| Cytidine | 1,000 | 7.75E-03 | 1.10E-01 |

ULOQ, pper limit of quantitation

Suppl.Table 7. Carry-over performance

| **Analyte** | **10x ULOQ**  **(nM)** | **LLOQ**  **(nM)** | **% of LLOQ** |
| --- | --- | --- | --- |
| Thymidine | 100,000 | 100 | 0.43 |
| Deoxyguanosine | 100,000 | 100 | 0.40 |
| Deoxyadenosine | 100,000 | 100 | 0.59 |
| Deoxycytidine | 100,000 | 100 | 0.50 |
| Deoxyuridine | 10,000 | 10 | 0.85 |
| Uridine | 10,000 | 10 | 0.31 |
| Guanosine | 1,000 | 1 | 0.64 |
| Adenosine | 1,000 | 1 | 0.44 |
| Cytidine | 1,000 | 1 | 0.74 |

LLOQ, Lower limit of quantitation

ULOQ, upper limit of quantitation

Suppl.Table 8. Stability of deoxyuridine under varying conditions.

| **Sample** | **Storage**  **Condition** | **Concentration**  **(nM)** | **Stability**  **(%)** | **CV**  **(%)** | **Replicates**  **(N)** |
| --- | --- | --- | --- | --- | --- |
| **Stock**  **Solution** | 6 h ambient temp | 100 | 91.4 | 10.2 | 3 |
|  | 2 years -80 °C | 100,000 | 97.4 | 3.64 | 5 |
| **Quality**  **Controls** | 4 h ambient temp | 15 | 107.0 | 15.2 | 4 |
|  |  | 150 | 105.5 | 8.2 | 4 |
|  |  | 800 | 104.7 | 11.2 | 4 |
|  | 3 freeze-thaw cycles -80 °C | 15 | 106.4 | 15.7 | 4 |
|  |  | 150 | 111.9 | 17.5 | 4 |
|  |  | 800 | 106.9 | 11.5 | 4 |
|  | 12 months at -80 °C | 15 | 88.7 | 9.3 | 4 |
|  |  | 150 | 93.0 | 14.0 | 4 |
|  |  | 800 | 95.0 | 6.3 | 4 |
|  | 72 h reinjection in autosampler at 4 °C | 10 | 110.3 | 10.1 | 6 |
|  |  | 15 | 110.2 | 5.4 | 6 |
|  |  | 150 | 104.0 | 3.6 | 6 |
|  |  | 800 | 100.9 | 6.7 | 6 |

10 nM: Lower limit of quantitation

15nM: Low QC

150 nM: Mid QC

800 nM: High QC

Suppl.Table 9. Stability of uridine under varying conditions.

| **Sample** | **Storage**  **Condition** | **Concentration**  **(nM)** | **Stability**  **(%)** | **CV**  **(%)** | **Replicates**  **(N)** |
| --- | --- | --- | --- | --- | --- |
| **Stock**  **Solution** | 6 h ambient temp | 100 | 90.8 | 5.6 | 3 |
|  | 2 years -80 °C | 100,000 | 96.8 | 4.7 | 5 |
| **Quality**  **Controls** | 4 h ambient temp | 15 | 109.0 | 11.9 | 4 |
|  |  | 150 | 108.9 | 5.4 | 4 |
|  |  | 800 | 109.5 | 2.3 | 4 |
|  | 3 freeze-thaw cycles -80 °C | 15 | 111.0 | 7.0 | 4 |
|  |  | 150 | 110.5 | 6.3 | 4 |
|  |  | 800 | 110.5 | 4.8 | 4 |
|  | 12 months at -80 °C | 15 | 98.6 | 11.9 | 4 |
|  |  | 150 | 107.6 | 11.1 | 4 |
|  |  | 800 | 106.2 | 9.6 | 4 |
|  | 72 h reinjection in autosampler at 4 °C | 10 | 98.6 | 8.9 | 6 |
|  |  | 15 | 97.0 | 6.2 | 6 |
|  |  | 150 | 99.9 | 2.5 | 6 |
|  |  | 800 | 100.9 | 2.4 | 6 |

10 nM: Lower limit of quantitation

15nM: Low QC

150 nM: Mid QC

800 nM: High QC

Suppl.Table 10. Stability of guanosine under varying conditions.

| **Sample** | **Storage**  **Condition** | **Concentration**  **(nM)** | **Stability**  **(%)** | **CV**  **(%)** | **Replicates**  **(N)** |
| --- | --- | --- | --- | --- | --- |
| **Stock**  **Solution** | 6 h ambient temp | 10 | 96.0 | 1.6 | 3 |
|  | 2 years -80 °C | 10,000 | 104.0 | 3.0 | 5 |
| **Quality**  **Controls** | 4 h ambient temp | 1.5 | 111.5 | 13.0 | 4 |
|  |  | 15 | 112.5 | 5.3 | 4 |
|  |  | 80 | 109.4 | 4.6 | 4 |
|  | 3 freeze-thaw cycles -80 °C | 1.5 | 109.3 | 12.1 | 4 |
|  |  | 15 | 110.7 | 6.2 | 4 |
|  |  | 80 | 108.8 | 5.0 | 4 |
|  | 12 months at -80 °C | 1.5 | 107.1 | 5.1 | 4 |
|  |  | 15 | 110.0 | 9.7 | 4 |
|  |  | 80 | 104.0 | 7.6 | 4 |
|  | 72 h reinjection in autosampler at 4 °C | 1.0 | 103.6 | 3.0 | 6 |
|  |  | 1.5 | 108.9 | 8.2 | 6 |
|  |  | 15 | 100.6 | 4.8 | 6 |
|  |  | 80 | 99.0 | 1.7 | 6 |

1.0 nM: Lower limit of quantitation

1.5 nM: Low QC

15 nM: Mid QC

80 nM: High QC

Suppl.Table 11. Stability of adenosine under varying conditions.

| **Sample** | **Storage**  **Condition** | **Concentration**  **(nM)** | **Stability**  **(%)** | **CV**  **(%)** | **Replicates**  **(N)** |
| --- | --- | --- | --- | --- | --- |
| **Stock**  **Solution** | 6 h ambient temp | 10 | 92.0 | 4.2 | 3 |
|  | 2 years -80 °C | 10,000 | 88.9 | 2.8 | 5 |
| **Quality**  **Controls** | 4 h ambient temp | 1.5 | 113.8 | 5.8 | 4 |
|  |  | 15 | 113.9 | 4.1 | 4 |
|  |  | 80 | 112.8 | 4.0 | 4 |
|  | 3 freeze-thaw cycles -80 °C | 1.5 | 112.4 | 6.5 | 4 |
|  |  | 15 | 114.1 | 6.7 | 4 |
|  |  | 80 | 113.8 | 4.8 | 4 |
|  | 12 months at -80 °C | 1.5 | 107.3 | 13.0 | 4 |
|  |  | 15 | 101.7 | 12.5 | 4 |
|  |  | 80 | 98.6 | 5.8 | 4 |
|  | 72 h reinjection in autosampler at 4 °C | 1.0 | 102.3 | 6.2 | 6 |
|  |  | 1.5 | 109.1 | 7.1 | 6 |
|  |  | 15 | 99.6 | 2.0 | 6 |
|  |  | 80 | 95.0 | 5.6 | 6 |

1.0 nM: Lower limit of quantitation

1.5 nM: Low QC

15 nM: Mid QC

80 nM: High QC

Suppl.Table 12. Stability of cytidine under varying conditions.

| **Sample** | **Storage**  **Condition** | **Concentration**  **(nM)** | **Stability**  **(%)** | **CV**  **(%)** | **Replicates**  **(N)** |
| --- | --- | --- | --- | --- | --- |
| **Stock**  **Solution** | 6 h ambient temp | 10 | 92.2 | 2.2 | 3 |
|  | 2 years -80 °C | 10,000 | 94.0 | 2.6 | 5 |
| **Quality**  **Controls** | 4 h ambient temp | 1.5 | 112.8 | 17.4 | 4 |
|  |  | 15 | 114.0 | 4.7 | 4 |
|  |  | 80 | 108.4 | 5.4 | 4 |
|  | 3 freeze-thaw cycles -80 °C | 1.5 | 107.2 | 10.8 | 4 |
|  |  | 15 | 114.3 | 7.1 | 4 |
|  |  | 80 | 111.3 | 7.2 | 4 |
|  | 12 months at -80 °C | 1.5 | 112.3 | 8.6 | 4 |
|  |  | 15 | 107.9 | 14.2 | 4 |
|  |  | 80 | 113.7 | 8.6 | 4 |
|  | 72 h reinjection in autosampler at 4 °C | 1.0 | 98.7 | 6.1 | 6 |
|  |  | 1.5 | 108.0 | 14.6 | 6 |
|  |  | 15 | 101.1 | 3.4 | 6 |
|  |  | 80 | 100.1 | 2.2 | 6 |

1.0 nM: Lower limit of quantitation

1.5 nM: Low QC

15 nM: Mid QC

80 nM: High QC

Suppl.Table 13. Stability of thymidine under varying conditions.

| **Sample** | **Storage**  **Condition** | **Concentration**  **(nM)** | **Stability**  **(%)** | **CV**  **(%)** | **Replicates**  **(N)** |
| --- | --- | --- | --- | --- | --- |
| **Stock**  **Solution** | 6 h ambient temp | 1,000 | 98.4 | 4.2 | 3 |
|  | 2 years -80 °C | 1,000,000 | 100.7 | 3.1 | 5 |
| **Quality**  **Controls** | 4 h ambient temp | 150 | 112.8 | 12.6 | 4 |
|  |  | 1,500 | 111.0 | 7.0 | 4 |
|  |  | 8,000 | 107.6 | 4.8 | 4 |
|  | 3 freeze-thaw cycles -80 °C | 150 | 108.6 | 12.4 | 4 |
|  |  | 1,500 | 112.9 | 7.3 | 4 |
|  |  | 8,000 | 108.3 | 7.2 | 4 |
|  | 12 months at -80 °C | 150 | 93.5 | 10.3 | 4 |
|  |  | 1,500 | 98.9 | 12.0 | 4 |
|  |  | 8,000 | 104.6 | 7.8 | 4 |
|  | 72 h reinjection in autosampler at 4 °C | 100 | 103.9 | 5.1 | 6 |
|  |  | 150 | 100.2 | 3.6 | 6 |
|  |  | 1,500 | 100.1 | 1.6 | 6 |
|  |  | 8,000 | 94.2 | 7.3 | 6 |

100 nM: Lower limit of quantitation

150 nM: Low QC

1,500 nM: Mid QC

8,000 nM: High QC

Suppl.Table 14. Stability of deoxyguanosine under varying conditions.

| **Sample** | **Storage**  **Condition** | **Concentration**  **(nM)** | **Stability**  **(%)** | **CV**  **(%)** | **Replicates**  **(N)** |
| --- | --- | --- | --- | --- | --- |
| **Stock**  **Solution** | 6 h ambient temp | 1,000 | 92.3 | 4.3 | 3 |
|  | 2 years -80 °C | 1,000,000 | 100.3 | 3.5 | 5 |
| **Quality**  **Controls** | 4 h ambient temp | 150 | 107.4 | 10.2 | 4 |
|  |  | 1,500 | 113.9 | 3.8 | 4 |
|  |  | 8,000 | 104.1 | 1.7 | 4 |
|  | 3 freeze-thaw cycles -80 °C | 150 | 109.5 | 11.5 | 4 |
|  |  | 1,500 | 113.3 | 4.3 | 4 |
|  |  | 8,000 | 109.0 | 6.0 | 4 |
|  | 12 months at -80 °C | 150 | 113.1 | 4.3 | 4 |
|  |  | 1,500 | 106.7 | 13.8 | 4 |
|  |  | 8,000 | 109.0 | 4.2 | 4 |
|  | 72 h reinjection in autosampler at 4 °C | 100 | 104.1 | 5.0 | 6 |
|  |  | 150 | 104.4 | 2.6 | 6 |
|  |  | 1,500 | 103.1 | 2.6 | 6 |
|  |  | 8,000 | 99.6 | 2.4 | 6 |

100 nM: Lower limit of quantitation

150 nM: Low QC

1,500 nM: Mid QC

8,000 nM: High QC

Suppl.Table 15. Stability of deoxyadenosine under varying conditions.

| **Sample** | **Storage**  **Condition** | **Concentration**  **(nM)** | **Stability**  **(%)** | **CV**  **(%)** | **Replicates**  **(N)** |
| --- | --- | --- | --- | --- | --- |
| **Stock**  **Solution** | 6 h ambient temp | 1,000 | 92.2 | 3.3 | 3 |
|  | 2 years -80 °C | 1,000,000 | 95.3 | 2.4 | 5 |
| **Quality**  **Controls** | 4 h ambient temp | 150 | 108.0 | 9.2 | 4 |
|  |  | 1,500 | 108.6 | 6.4 | 4 |
|  |  | 8,000 | 109.5 | 3.3 | 4 |
|  | 3 freeze-thaw cycles -80 °C | 150 | 108.8 | 8.1 | 4 |
|  |  | 1,500 | 111.5 | 5.6 | 4 |
|  |  | 8,000 | 107.8 | 4.3 | 4 |
|  | 12 months at -80 °C | 150 | 101.9 | 4.7 | 4 |
|  |  | 1,500 | 95.7 | 11.9 | 4 |
|  |  | 8,000 | 108.5 | 8.1 | 4 |
|  | 72 h reinjection in autosampler at 4 °C | 100 | 101.6 | 4.3 | 6 |
|  |  | 150 | 99.5 | 3.9 | 6 |
|  |  | 1,500 | 101.3 | 3.3 | 6 |
|  |  | 8,000 | 99.7 | 4.9 | 6 |

100 nM: Lower limit of quantitation

150 nM: Low QC

1,500 nM: Mid QC

8,000 nM: High QC

Suppl.Table 16. Stability of deoxycytidine under varying conditions.

| **Sample** | **Storage**  **Condition** | **Concentration**  **(nM)** | **Stability**  **(%)** | **CV**  **(%)** | **Replicates**  **(N)** |
| --- | --- | --- | --- | --- | --- |
| **Stock**  **Solution** | 6 h ambient temp | 1,000 | 91.6 | 3.4 | 3 |
|  | 2 years -80 °C | 1,000,000 | 97.9 | 2.6 | 5 |
| **Quality**  **Controls** | 4 h ambient temp | 150 | 112.8 | 11.1 | 4 |
|  |  | 1,500 | 112.3 | 7.2 | 4 |
|  |  | 8,000 | 113.6 | 3.3 | 4 |
|  | 3 freeze-thaw cycles -80 °C | 150 | 110.8 | 7.6 | 4 |
|  |  | 1,500 | 113.9 | 7.4 | 4 |
|  |  | 8,000 | 112.7 | 6.0 | 4 |
|  | 12 months at -80 °C | 150 | 103.9 | 4.6 | 4 |
|  |  | 1,500 | 99.8 | 10.6 | 4 |
|  |  | 8,000 | 99.0 | 8.8 | 4 |
|  | 72 h reinjection in autosampler at 4 °C | 100 | 102.3 | 1.9 | 6 |
|  |  | 150 | 103.2 | 1.5 | 6 |
|  |  | 1,500 | 101.2 | 2.0 | 6 |
|  |  | 8,000 | 101.5 | 1.6 | 6 |

100 nM: Lower limit of quantitation

150 nM: Low QC

1,500 nM: Mid QC

8,000 nM: High QC

Suppl.Table 17. Representative rN and dN concentrations from WT and ΔUNG MEF cells exposed to 5 µM ceralasertib for 0, 4, and 8 h

|  | **Sample** | **dUrd**  **(nM)** | **Urd**  **(nM)** | **Gua**  **(nM)** | **Ade**  **(nM)** | **Cyt**  **(nM)** | **Thy**  **(nM)** | **dGua**  **(nM)** | **dAde**  **(nM)** | **dCyt**  **(nM)** |
| --- | --- | --- | --- | --- | --- | --- | --- | --- | --- | --- |
| **Treatments** | WT - 0h | 460 (28) | 54 (5) | 208 (9) | 37 (1) | 83 (5) | 23,567 (1076) | 17,033 (914) | 20,244 (1,450) | 14,722 (920) |
|  | WT - 4h | 513 (31) | 60 (4) | 224 (6) | 39 (3) | 89 (5) | 25,638 (1244) | 19,113 (1,110) | 23,325 (1,004) | 18,125 (1,613) |
|  | WT - 8h | 651 (60) | 59 (6) | 210 (10) | 38 (3) | 90 (10) | 28,089 (1838) | 21,567 (1,534) | 25,178 (1,717) | 19,300 (846) |
|  | ΔUNG - 0h | 629 (36) | 52 (4) | 204 (6) | 33 (2) | 74 (6) | 30,844 (2185) | 22,833 (1,267) | 26,489 (1,536) | 20,122 (1,449) |
|  | ΔUNG - 4h | 703 (67) | 52 (5) | 199 (9) | 31 (1) | 74 (9) | 31,700 (1859) | 23,967 (1,849) | 28,278 (1,930) | 21,289 (1,839) |
|  | ΔUNG - 8h | 724 (100) | 48 (5) | 202 (11) | 31 (2) | 68 (9) | 33,211 (1292) | 25,556 (1,240) | 29,056 (1,202) | 21,744 (1,555) |
| **Controls** | Water | ***BLQ*** | ***BLQ*** | ***BLQ*** | ***BLQ*** | ***BLQ*** | ***BLQ*** | ***BLQ*** | ***BLQ*** | ***BLQ*** |
|  | Water + Buffer | ***BLQ*** | ***BLQ*** | ***BLQ*** | ***BLQ*** | ***BLQ*** | ***BLQ*** | ***BLQ*** | ***BLQ*** | ***BLQ*** |
|  | Water + Degradase | ***BLQ*** | 26.7 | 182 | 14.7 | 30.3 | 219 | 198 | 207 | 177 |
|  | Water + Buffer + Degradase | ***BLQ*** | 54.7 | 220 | 41 | 67.7 | 218 | 196 | 205 | 168 |

Values are reported as mean (SD) from 3 biological replicates of 0, 4, and 8 hour samples

BLQ: Below limit of quantitation

# Supplementary Figures

Suppl.Fig. 1. Representative calibration curves and residuals (N=3/level) for rN analytes. A,B) Cyt (response = 0.0747 • conc + 0.0196; R^2^ = 0.998); C,D) Ade (response = 0.1520 • conc + 0.0440; R^2^ = 0.999); E,F) Gua (response = 0.0156 • conc + 0.0037; R^2^ = 0.996); G,H) Urd (response = 0.0027 • conc - 0.0003; R^2^ = 0.998). Calibration curves are depicted as response ratio versus nominal concentration and associated residuals (%) are representative of the back-calculated relative to the nominal concentrations versus the log-transformed concentration.

Suppl.Fig. 2. Representative calibration curves and residuals (N=3/level) for dN analytes. A,B) dUrd (response = 0.0041 • conc - 0.0013; R^2^ = 0.998); C,D) Thy (response = 0.0024 • conc + 0.0190; R^2^ = 0.998); E,F) dGua (response = 0.0025 • conc - 0.0034; R^2^ = 0.998); G,H) dAde (response = 0.0020 • conc + 0.0024; R^2^ = 0.999); I,J) dCyt (response = 0.0029 • conc + 0.0092; R^2^ = 0.999). Calibration curves are depicted as response ratio versus nominal concentration and associated residuals (%) are representative of the back-calculated relative to the nominal concentrations versus the log-transformed concentration.

Suppl.Fig. 3. Analytical carry-over of: A) Cyt B) Ade C) Gua D) Urd E) dUrd F) Thy G) dGua H) dAde I) dCyt described in serial blank injections following a spiked analyte sample 10x ULOQ. Data represented as mean percent analyte response relative to 10x ULOQ response ± SD from 3 independent runs.
